# Direct Georeferencing for the Images in an Airborne LiDAR System by Automatic Boresight Misalignments Calibration

**DOI:** 10.3390/s20185056

**Published:** 2020-09-05

**Authors:** Haichi Ma, Hongchao Ma, Ke Liu, Wenjun Luo, Liang Zhang

**Affiliations:** 1 School of Remote Sensing and Information Engineering, Wuhan University, Wuhan 430079, China; mahaichi@whu.edu.cn (H.M.); Liu_ke@whu.edu.cn (K.L.); yunitongzai@whu.edu.cn (W.L.); 2 Department of Oceanography, Dalhousie University, Halifax, NS B3H 4R2, Canada; 3 Faculty of Resources and Environmental Science, Hubei University, Wuhan 430062, China; zhangliang@hubu.edu.cn

**Keywords:** direct georeferencing, Calibration, digital camera, LiDAR

## Abstract

Airborne Light Detection and Ranging (LiDAR) system and digital camera are usually integrated on a flight platform to obtain multi-source data. However, the photogrammetric system calibration is often independent of the LiDAR system and performed by the aerial triangulation method, which needs a test field with ground control points. In this paper, we present a method for the direct georeferencing of images collected by a digital camera integrated in an airborne LiDAR system by automatic boresight misalignments calibration with the auxiliary of point cloud. The method firstly uses an image matching to generate a tie point set. Space intersection is then performed to obtain the corresponding object coordinate values of the tie points, while the elevation calculated from the space intersection is replaced by the value from the LiDAR data, resulting in a new object point called Virtual Control Point (VCP). Because boresight misalignments exist, a distance between the tie point and the image point of VCP can be found by collinear equations in that image from which the tie point is selected. An iteration process is performed to minimize the distance with boresight corrections in each epoch, and it stops when the distance is smaller than a predefined threshold or the total number of epochs is reached. Two datasets from real projects were used to validate the proposed method and the experimental results show the effectiveness of the method by being evaluated both quantitatively and visually.

## 1. Introduction

Airborne laser scanning, also termed airborne Light Detection and Ranging (LiDAR), is an active remote sensing technique for acquiring 3D geospatial data over the Earth’s surface [1,2]. A typical airborne LiDAR system consists of a GPS (Global Positioning System), an IMU (Inertial Measurement Unit), and a laser scanner, with which a point cloud dataset encoding 3D coordinate values under a given geographic coordinate system can be generated [3]. The point cloud can be further processed to extract thematic information and geo-mapping products, such as manmade objects [4], stand-alone plants [5], DEM (Digital Elevation Model)/DTM (Digital Terrain Model) [6], etc. However, there are still many challenges in terms of object detection, extraction, and reconstruction by using the LiDAR dataset alone, because the point cloud provided by a LiDAR system is unstructured, irregularly spaced, and lacks spectral and textural information. Thus, a commercial airborne LiDAR system usually integrates a high-resolution metric digital camera, from which high-resolution aerial images can be collected while collecting point cloud data. The individual characteristics of LiDAR point cloud and image data are considered complementary [7]. They have been used to enhance the extraction of thematic information by fusing the two datasets for a variety of applications, such as buildings detection and reconstruction [8,9], land cover classification [10,11], road modeling [12,13], and tree species classification [14,15], to name but a few.

In photogrammetric applications, it is necessary to determine the geometric model of the sensing system before the collected images can be used for highly accurate measurement purposes. In traditional aerial photogrammetric mapping, the process begins with the determination of the IOEs (Interior Orientation Elements) and the EOEs (Exterior Orientation Elements) of the camera. IOEs are usually provided by the camera manufacturer [16]. This means that IOEs can be viewed as known variables during the photogrammetric processing. EOEs can be processed in two steps (relative and absolute orientation), but simultaneous methods (such as bundle adjustments) are now available in the majority of software packages [17]. A photogrammetric test field with highly redundant photo coverage such as 80% forward overlap and 60% side overlap and accurate ground control points (GCPs) are required in the simultaneous methods [18,19]. With the availability of the combination of GPS/IMU, direct georeferencing becomes possible because the EOEs can be derived from an integration of relative kinematic GPS positioning and IMU data by Kalman filtering, which is the case in an airborne LiDAR system integrated with a digital camera.

**Figure 1:**
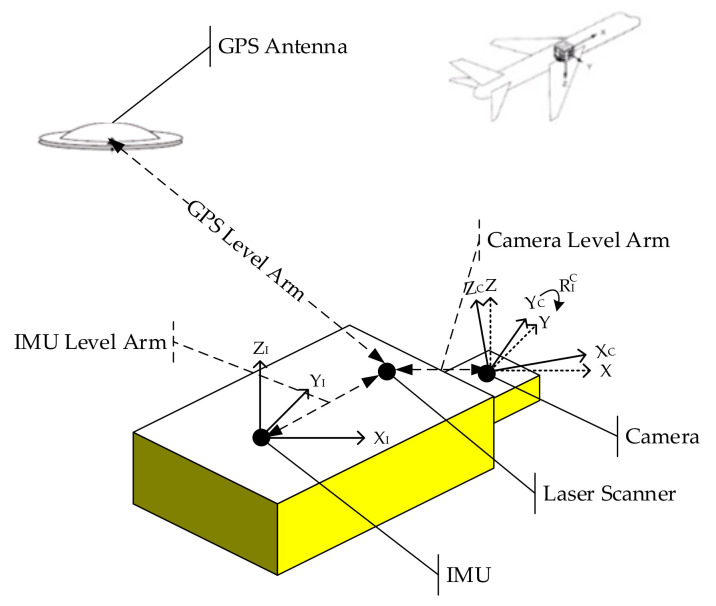
Positional Relationship between Laser Scanner, Camera, GPS, and IMU.

One of the prerequisites for direct georeferencing of images is the rigid connection between the camera and the IMU in order to keep a strict parallel between the image sensing frame and the IMU body frame, which is hard to achieve and may vary even within a given flight day [20]. Moreover, as the origin of the camera frame cannot be coincident with the projection center of the camera, and the GPS antenna will be on the top of the aircraft, the attitude and positional relation between camera and IMU, known as boresight errors/misalignments, must be determined before direct georeferencing of images can be performed, which includes the determination of three rotational angles and three lever arms, as shown in Figure 1. Level arms can be measured by traditional methods, such as direct measurement with ranging tools, close range photogrammetry [21], and accuracy within one centimeter can be achieved [22]. However, the measurements of the boresight misalignments are far more complicated compared to lever arm measurements because no direct methods exist. Conventionally, they are determined indirectly by using a reference block with known ground control points located within the project area or in a special test field, a process termed as system calibration, because it provides the calibration of other parameters such as focal length. Many research works have been conducted regarding direct georeferencing with the conventional method in the past two decades. Heier et al. [23] showed the postprocessing steps of DMC (Digital Metric Camera) image data to generate virtual central perspective images and gave an overview of the entire DMC calibration. Skaloud et al. [24] and Skaloud [25] conducted a study on the method of GPS/IMU integration to provide exterior orientation elements for direct georeferencing of airborne imagery with more reliability and better accuracy. The operational aspects of airborne mapping with GPS/IMU were analyzed and strategies for minimizing the effect of the hardware integration errors on the process for direct georeferencing were proposed. Heipke et al. [26] discussed the direct determination of the EOEs via the combination of GPS and IMU as a complete substitute for aerial triangulation. Jacobsen [27] discussed the direct georeferencing based on restoring the geometric relations of images in a chosen object coordinate system, and the possibility of avoiding using control points by direct sensor orientation with the combination of GPS and IMU. Mostafa et al. [28] argued that boresight misalignments calibration is one of the critical steps in direct georeferencing for geomapping purposes. They presented the experimental results of boresight misalignments calibration by using a software and checked the results with ground control points. Honkavaara [29,30] discussed block structures for calibration that significantly affected the cost and efficiency of the system calibration. The experiments indicated that boresight misalignments and the IOEs are the main factors influencing the final results. Jacobsen [31] investigated the direct and integrated sensor orientation based on the combination of relative kinematic GPS and IMU. The investigation showed the advantages of using direct sensor orientation for image georeferencing without ground control points and independent of block or strip configurations. Filho et al. [32] presented an in-flight calibration method for multi-head camera systems, and the applications of direct georeferencing were evaluated.

Though intensively adopted in practice, traditional system calibration shows the following drawbacks: Firstly, the environmental conditions such as temperature, humidity, etc. between the test field and mapping areas may dramatically differ. Therefore, the camera geometry during operation may also change relative to the situation in the test filed due to changes in environmental conditions [33,34]. Secondly, establishing a new test field for every mapping project and collecting large numbers of ground control points is expensive and sometimes impractical. On the other hand, airborne LiDAR systems deliver direct dense 3D measurements of object surface at a high rate of accuracy [7,16]. Moreover, continued improvements in the performance and accuracies of LiDAR systems in recent years have enabled the use of LiDAR data as a source of control information suitable for photogrammetric applications. Different methods have been tested and implemented for integrating LiDAR and photogrammetric data in performing aerial triangulation or determining the boresight misalignments for direct georeferencing, as will be reviewed in the following.

Delara et al. [35] presented a method to perform the bundle block adjustment using aerial images and laser scanner data. In the method, LiDAR control points were extracted from LiDAR intensity images for determining the exterior orientation elements of a low-cost digital camera. Habib et al. [36,37] utilized linear features derived from LiDAR data as control information for image georeferencing. However, a large number of linear features with good spatial distribution are needed to achieve high accuracy. Kwak et al. [38] proposed using the centroid of the plane roof surface of a building as control information for estimating exterior orientation elements of aerial imagery and registering the aerial imagery relative to the aerial LiDAR data. In the method, the centroid of the plane roof is extracted from aerial imagery by using the Canny Edge Detector and from aerial LiDAR data by using Local Maxima Filtering. Liu et al. [39] presented a method for utilizing LiDAR intensity images to collect high accuracy ground coordinates of GCPs for aerial triangulation process. Yastikli et al. [40] investigated the feasibility of using LiDAR data for in situ calibration of the digital camera. In addition, the determination of attitude and positional relationship between digital camera and IMU was also discussed. Mitishita et al. [41] presented a method of georeferencing photogrammetric images using LiDAR data. The method applied the centroids of rectangular building roofs as control points in the photogrammetric procedure. Ding et al. [42] utilized the vertical vanishing point in an aerial image and the corner points of the roof edge from the point cloud to estimate the pitch and roll of the cameras rotation angles. Based on Ding’s study, Wang and Neumann [43] introduced a new feature 3CS (three connected segments) to replace the vanishing point to optimize the method. Each 3CS has three segments connected into a chain. Wildan et al. [44] utilized control points derived from LiDAR data to perform the aerial triangulation of a large photogrammetric block of analogue aerial photographs. According to the authors, the mapping has achieved the national standard of cartographic accuracy for the 1:50,000 scale mapping. Chen et al. [45] proposed a new method for boresight misalignments calibration of the digital camera integrated in an airborne LiDAR system without ground control points. In the calibration, tie points in overlapping areas are selected manually, and the ground points corresponding to these points are calculated using a multi-baseline space intersection and DEM elevation constraints. Gneeniss [46] and Gneeniss et al. [16] conducted studies on cross-calibrate aerial digital cameras via the use of complementary LiDAR data. The effect of the number and spatial distribution of LiDAR control points to perform aerial triangulation of large photogrammetric blocks was investigated as well.

Direct georeferencing of images based on LiDAR point cloud was also provided by commercial software Terrasolid in the TMatch module. In this module, a filter is used firstly to obtain ground points from point cloud, while a large number of tie points of the images are manually selected. The optimal camera misalignment values (new heading, roll, and pitch) are calculated using the tie points from the overlapping images and their corresponding ground points from the LiDAR point cloud. However, image points and LiDAR-derived ground points are matched manually, where artificial errors are inevitable, and matching is impractical when the surveying area is large.

The objective of this study is to introduce a new automatic boresight misalignments calibration method for direct georeferencing of images collected by a digital camera integrated in an airborne LiDAR system. Because the three lever arms can be accurately measured, we focus on the determination of the three rotational angles by using the LiDAR point cloud as auxiliary data. In contrast to the methods presented in previous literature, the in situ camera calibration focuses on using VCPs (Virtual Control Points—defined in following section) and a small sub-block of images selected from the entire block covering the surveying area. The method establishes the error equation by minimizing the distances between the initially selected tie points in the image space and the image points corresponding to VCPs by space resection. The main advantages of the method can be summarized as follows: Firstly, no dedicated calibration test fields, or even ground control points, are needed. Secondly, the whole procedure is fully automatic, from the extraction of tie points to the calibration of the boresight misalignments. This is of particular importance when the airborne LiDAR system is employed to collect data for rapid response to natural disasters, such as earthquake relief efforts. Finally, the accuracy of the georeferenced images is high enough for many geospatial applications, as shown in the experimental results.

## 2. Materials and Methods

### 2.1. Sample Materials

**Figure 2:**
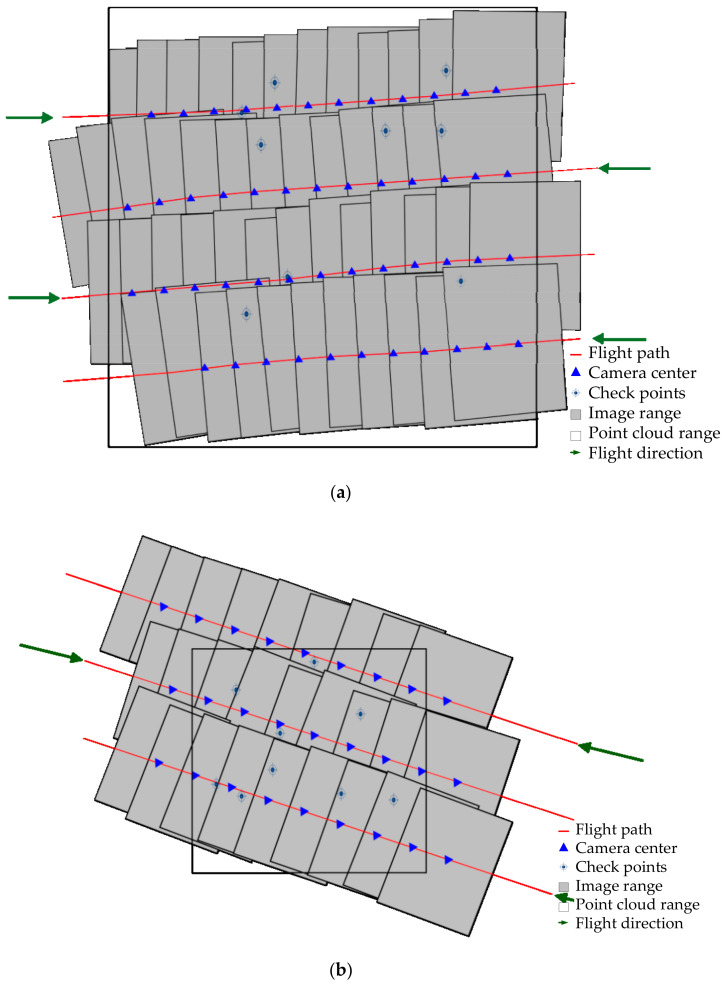
Block layout configuration: (**a**) Dataset 1; and (**b**) Dataset 2.

Two datasets were used to test the effectiveness of the proposed method. The first dataset was acquired in a suburb of Xi’an, Shaanxi province, China, and consists of LiDAR point cloud and aerial images. Images were collected by Leica RCD105 and point cloud was acquired by Leica ALS60. Specifications of the equipment and the data are listed in Table 1. Figure 2a shows the flight direction of the strips. The selected sub-set this dataset covers extends approximately 3.26 km in the east–west and 3.34 km in the north–south direction. The second dataset came from Ningbo, Zhejiang Province, China, and also consists of point cloud and optical images. Although both of the LiDAR systems adopted to collect the two datasets were manufactured by Leica Geosystem, specifications of the equipment and data are different (see Table 2 and Figure 2b for more details of the second dataset).

Outliers in the point cloud were filtered before it was input to the calibration process. This can be achieved either by a commercial software or open source libraries such as PCL (Point Cloud Library) [47].

### 2.2. Methods

#### 2.2.1. Overview of the Methods

We first define the concept of virtual control points. For an airborne LiDAR system integrated with a digital camera, assuming that boresight misalignments have been corrected and lever arms were accurately measured, select a sub-block of images where two adjacent images meet the requirement of at least 60% forward overlap. Then, for any pair of tie points in the two images, their object coordinates can be derived from the collinearity equations with the exterior orientation elements provided by GPS/IMU combined navigation system, or synonymously termed as Positioning and Orientation System (POS) in the airborne LiDAR community, which are accurate enough because boresight misalignments were corrected and lever arms were accurately measured. Thus, a given pair of tie points 

**Figure 3:**
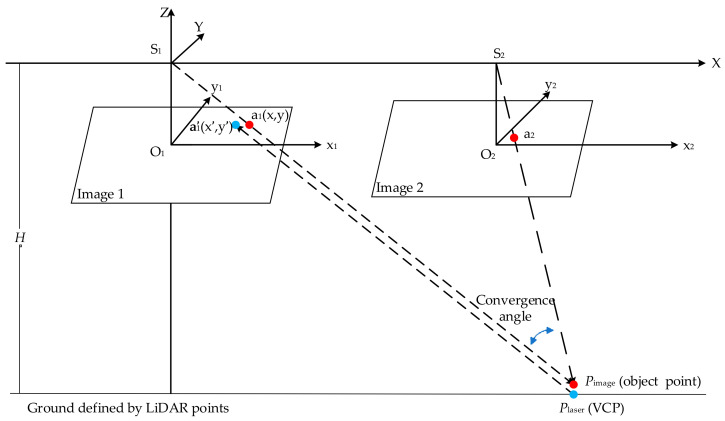
The distance between $a_{1}$ and $a_{1}^{\prime }\;$: $S_{1}$ and $S_{2}$ are the spatial positions of the projection center of the camera at the moment of acquiring Images 1 and 2. H is the altitude of the aircraft.

$a_{1}$ and $a_{2}$ (see Figure 3), determine a corresponding object point, denoted by *P*_image_ with object coordinates (*X*, *Y*, *Z*_image_) under a given object coordinate system. If the adjacent images cover a flat area, then the elevation value of the area shall be a constant, which can be derived from LiDAR point cloud and be denoted by *Z*_laser_. Currently, vertical accuracy better than several centimeters can be achieved in flat areas by a commercial airborne LiDAR system [48]; thus, in most topographic mapping applications, the elevation value from the point cloud can be treated as the true value. If $a_{1}$ and $a_{2}$ were accurately matched, the object point *P*_image_ should lie in the flat plane. However, due to the existence of systematic errors, such as boresight misalignments and other random errors, *P*_image_ will be off the plane; that is, its elevation value *Z*_image_ can be significantly greater or less than the elevation value derived from point cloud *Z*_laser_. Replace *Z*_image_ by *Z*_laser_ to create an object point with object coordinates (*X*, *Y*, *Z*_laser_), which is defined as a Virtual Control Point (VCP).

Our method begins with image matching to generate a set of tie points. For each pair of tie points, a VCP can be derived as described above. As shown in Figure 3, denote the image coordinates of tie point $a_{1}$ by x and y and denote its VCP by *P*_laser_. Reproject *P*_laser_ onto Image 1 by using collinearity equations, and then an image point $a_{1}^{\prime }$ can be obtained. If *Z*_laser_ = *Z*_image_, then $a_{1}$ and $a_{1}^{\prime }$ are identical. However, due to the existence of boresight misalignments and other random errors, the distance between a_1_ and $a_{1}^{\prime }$ cannot be neglected. The total distance between all of the tie points and their corresponding points that are derived from VCPs is calculated. Minimizing the total distance by iteratively correcting the boresight misalignments is the core idea of the proposed method.

**Figure 4:**
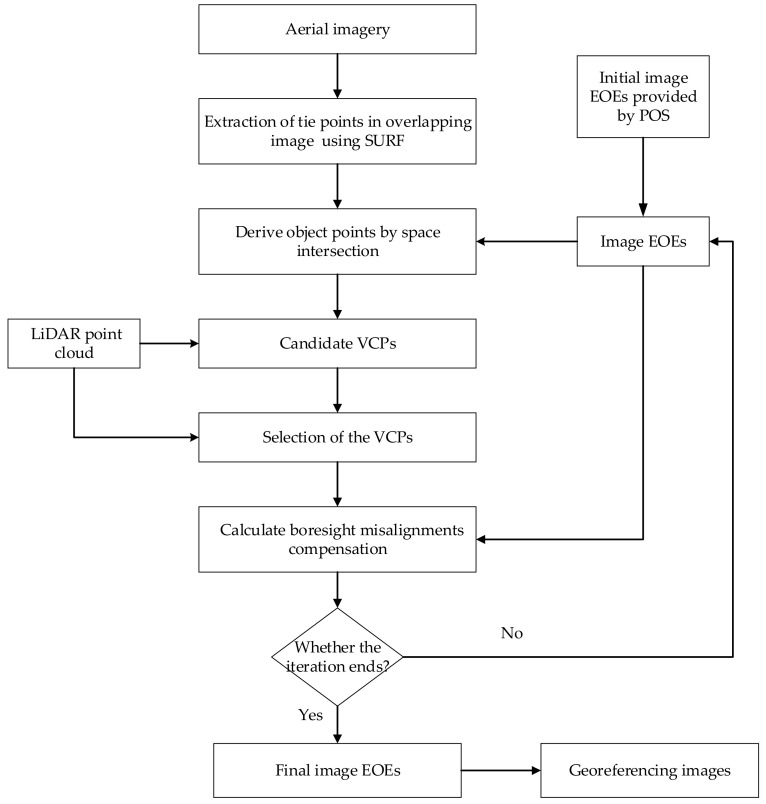
Flowchart of the proposed method.

The general workflow of the method is illustrated in Figure 4. The main steps include:

1. Select sub-block images collected over a relatively flat area from image set and extract tie points in the overlapping areas of the sub-block images using a Speed-Up Robust Features (SURF) algorithm [49].
2. For each pair of tie points, the object point can be derived by space intersection.
3. Replace the elevation values of object points by those derived from LiDAR point cloud. These new points are called VCPs.
4. An automatic VCP selection procedure is designed to perform various assessments to guarantee high quality VCPs can be selected.
5. Adjustment equation is established to perform boresight misalignments compensation based on the combination of the VCP set and collinearity equations.
6. Repeat Steps 2–5 until the total distance between all tie points and their corresponding points that are derived from VCPs remains stable in the iteration or the maximum iteration has been reached.

In the following subsection, detailed explanations are stated, and key equations and formulas are provided.

#### 2.2.2. Detection and Matching of Tie Points in Overlapping Images Using SURF Algorithm

Detection and matching tie points in overlapping image areas is the first step in the method. The removal of boresight misalignments is largely affected by the accuracy of tie points. Thanks to the progress of image matching techniques [49,50,51,52,53,54], automatic tie points extraction with high accuracy becomes operational.

Although there are many image matching algorithms available, Speed-Up Robust Feature (SURF) [49] is adopted in this study. It is a robust local feature point descriptor, which consists of three major stages: (1) interest point detection; (2) orientation assignment; and (3) interest point description. In the first stage, potential interest points are identified by scanning the image over location and scale. This is effectively achieved by the Hessian matrix using speckle detection. In the second stage, the dominant orientation for each interest point is identified based on its local image patch. The third stage builds a local image descriptor for each interest point, based upon the image gradients in its local neighborhood. This is followed by establishing correspondences with interest points of different images to obtain connection points.

The performance of the SURF algorithm is similar to Scale Invariant Feature Transform (SIFT) [51] in many respects, including robustness to lighting, blur, and perspective distortion. However, it improves computational efficiency by using integral images, Haar wavelet transforms, and approximate Hessian matrix operations. In direct georeferencing, the faster the calculation speed is, the higher the value is in practical survey tasks, without sacrificing an accurate performance. Due to its high efficiency, accuracy, and robustness, we use the SURF algorithm to extract image connection points. In addition, in the process of feature point registration, as the image has been positioned using the initial elements provided by the POS system, only the feature points in the center *S*-scale window where the feature points are located are paired. This method can effectively improve computing efficiency, as shown in the experiment section of the study.

For each pair of tie points, the object point is derived by space intersection where exterior orientation elements are provided directly by the POS system without the correction of boresight misalignments.

The fundamental of space intersection is that conjugate light rays meet in the object space. The angle between two conjugate rays is defined as convergence angle in [55] (see Figure 3), and the authors showed that the positional accuracy decreases dramatically when the convergence angle is less than 14.6 degrees. Therefore, if the convergence angle is less than this threshold, the object point obtained by space intersection is labeled as a non-candidate; otherwise, it is labeled as a candidate from which the VCPs will be selected. The convergence angle can be calculated according to Equation (1).

(1) $$\cos\varphi =\frac{\vec{S_{1}a_{1}}.\vec{S_{2}a_{2}}}{\left|\vec{S_{1}a_{1}}\right|.\left|\vec{S_{2}a_{2}}\right|}=\frac{\left(X_{s_{1}}-X_{a_{1}}\right).\left(X_{s_{2}}-X_{a_{2}}\right)+\left(Y_{s_{1}}-Y_{a_{1}}\right).\left(Y_{s_{2}}-Y_{a_{2}}\right)+\left(Z_{s_{1}}-Z_{a_{1}}\right).\left(Z_{s_{2}}-Z_{a_{2}}\right)}{\sqrt{{\left(X_{s_{1}}-X_{a_{1}}\right)}^{2}+{\left(Y_{s_{1}}-Y_{a_{1}}\right)}^{2}+{\left(Z_{s_{1}}-Z_{a_{1}}\right)}^{2}}.\sqrt{{\left(X_{s_{2}}-X_{a_{2}}\right)}^{2}+{\left(Y_{s_{2}}-Y_{a_{2}}\right)}^{2}+{\left(Z_{s_{2}}-Z_{a_{2}}\right)}^{2}}}$$

where S_1_ and S_2_ are the center positions of the camera at the moment of acquiring Images 1 and 2, respectively; and $a_{1}$ and $a_{2}$ are a pair of tie points.

#### 2.2.3. Selection of the VCPs

A large number of tie points can be detected from two overlapping images after the tie points detection step. Theoretically, the same amount of object points corresponding to tie points can be derived by space intersection.

**Figure 5:**
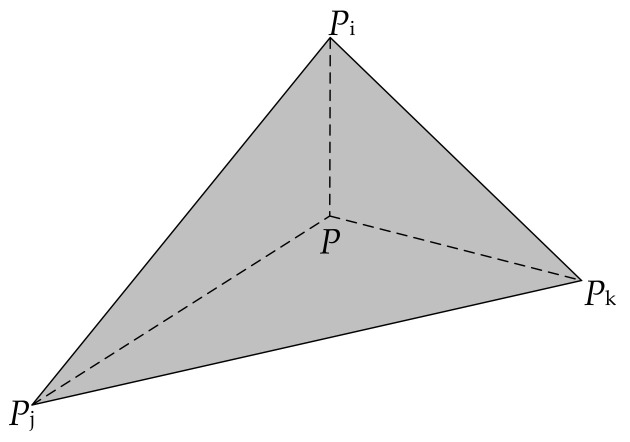
A VCP point *P* inside a triangle formed by *P*_i_, *P*_j_, and *P*_k._

The first step of VCP selection is the replacement of the elevation values of object points by those derived from LiDAR point cloud. This is a relatively simple step, which mainly involves searching the point cloud via the x and y coordinates of the object points and then replacing their elevation values by interpolation; a new point set called candidate VCPs is obtained, from which the VCPs will be selected. In detail, the step proceeds as follows: the point cloud is projected onto the x–y plane, and then a 2D triangulated irregular network (TIN) is constructed based on the projected points. For a given object point (*X*, *Y*, *Z*), it is determined which triangle the candidate falls into. If it happens to be positioned at one vertex of a triangle, then the *Z* value is replaced by that vertex. Otherwise, it is estimated by inverse distance weighted interpolation with elevation values of the three vertices as known points to the interpolation (see Figure 5).

Importantly, as defined in Section 2.2.1, a VCP should be located in a flat patch of a building or a terrain surface so that the elevation values of the object points in it are insensitive to planar positions; that is, given two object points *P*_1_ (*X*_1_, *Y*_1_, *Z*_1_) and P_2_ (*X*_2_, *Y*_2_, *Z*_2_) in a flat patch, then elevation values *Z*_1_ ≈ *Z*_2_ regardless of where *P*_1_ and *P*_2_ are located in the patch. This is a very important constraint of the proposed method because it makes the method insensitive to the planar errors of the object points, which is mainly caused by inaccurate image matching. Thus, several criteria for VCP selection have been designed (see below) in order to guarantee that all the VCPs are located in flat or approximately flat patches and that all of them are reliable.

Planarity: Flat patches, roads, playgrounds, flat building roofs, etc. are the main surfaces where VCPs can be located. Planarity is measured by finding the best-fit plane for the LiDAR points in a window with a predefined size centered at a VCP. At least four points are required for a plane fitting (Equation (2)), thus the window size and the density of the point cloud are two key parameters for the plane fitting. In [56], a more specific definition of density for building recognition is proposed and it is pointed out that an average density of 1 point/m^2^ can detect a building roof with size 2.8 m × 2.8 m. Thus, considering that in most practical cases the average density is higher than 1 point/m^2^, a 3 m × 3 m size window is adopted in the study.

(2) A×x+B×y+C×z+D=0

A threshold of 0.2 m was set to check if a candidate VCP is located in the fitted plane: if the distance from it to the fitted plane less than 0.2 m, then it remains in the VCP set; otherwise, it is labeled as non-VCP.

**Figure 6:**
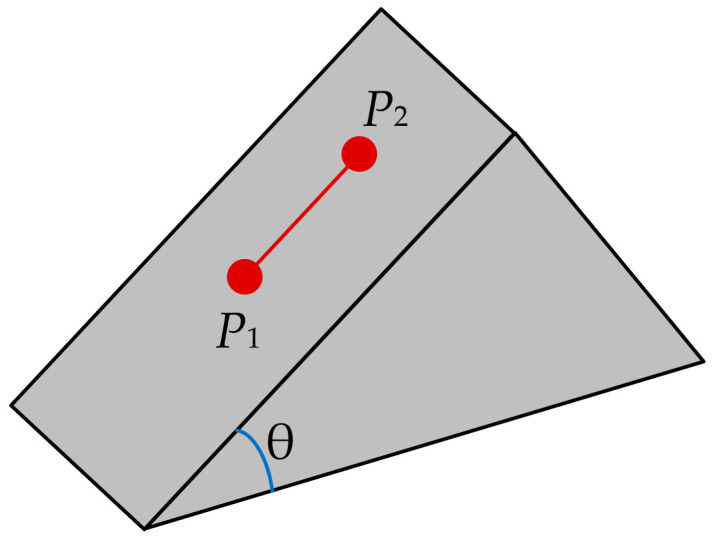
Elevation difference on slope.

Slope tolerance: An absolute flat patch is almost nonexistent in real situations, thus it is valuable to study the elevation variation with the change of the slope of a planar patch along the steepest ascend or descend direction (see Figure 6). A simple calculation shows that, when the distance between *P*_1_ and *P*_2_ is 1 m, then the elevation difference is 14 cm when the slope is 8 degrees. Bearing in mind that most commercial LiDAR systems achieve vertical accuracy better than 14 cm, planar patches with slopes less than or equal to 8 degrees were classified as flat patches.

Reliability checking: Theoretically, two stereo images reconstruct the 3D terrain of the area covered by the overlapping part of the images. Presently, however, overlapping more than 80% is common in practice thanks to the development of digital cameras, which allow more than two images to overlap the same area. Multiple stereo images not only improve the accuracy of the object coordinates of tie points, but also provide a method for reliability checking of the candidate VCPs. This is because the errors along the *x*-axis of an object point intersected by two rays cannot be readily detected, which can be overcome by the intersection of more than two rays [57]. This check is not required because VCPs are selected from flat patches, which means the planar errors are insensitive to the elevation values and that the process of boresight misalignments correction is an iterative one, leaving the planar errors to be corrected in the iterations.

#### 2.2.4. Boresight Misalignments Calibration

**Figure 7:**
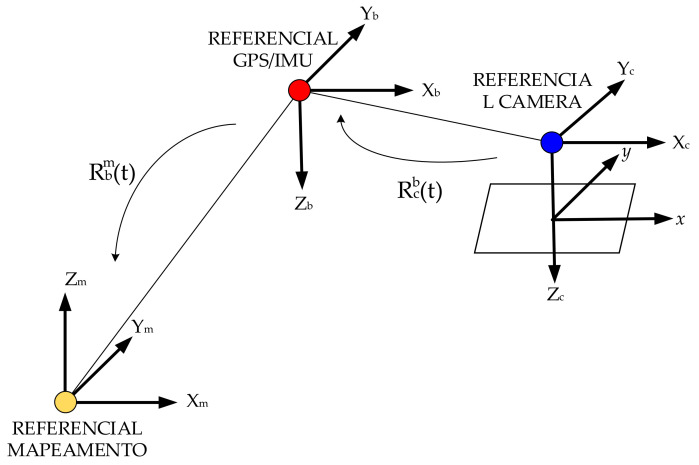
Coordinate transformation for direct georeferencing.

As stated in the introduction, there is much literature available regarding boresight misalignments calibration. A new calibration method was proposed by Baumker and Heimes in 2002 [58], in which the boresight misalignments were contained in a misalignments matrix, which, with other transformation matrices, forms a complete transformation matrix from terrain to image system. An adjustment equation was then formed for each photo, followed by the establishment of a total normal equation including all measurements. Straying from Baumker’s method, our model is constrained by the minimization of the total distance between tie points and image points calculated from VCPs by space resection, thus the transformation matrix containing the boresight misalignments is included in the colinear equations where boresight misalignments are treated as extra rotations around three axes alongside three exterior orientation elements, φ,ω,and k. Figure 7 shows the coordinate transformation for direct georeferencing.

The proposed method is based on the traditional collinear equations, which relate the relationship among image pixels coordinates, ground points coordinates, and photographic center as shown in the following equations:

(3) $$\left[\begin{array}{c} X\\ Y\\ Z \end{array}\right]=\lambda R_{b}^{m}\left(\varphi ,\omega ,\kappa \right)\left[\begin{array}{c} x\\ y\\ -f \end{array}\right]+\left[\begin{array}{c} X_{S}\\ Y_{S}\\ Z_{S} \end{array}\right]=\lambda \left[\begin{array}{ccc} a_{1} & a_{2} & a_{3}\\ b_{1} & b_{2} & b_{3}\\ c_{1} & c_{2} & c_{3} \end{array}\right]\left[\begin{array}{c} x\\ y\\ -f \end{array}\right]+\left[\begin{array}{c} X_{S}\\ Y_{S}\\ Z_{S} \end{array}\right],$$

where

(4) $$\left[\begin{array}{ccc} a_{1} & a_{2} & a_{3}\\ b_{1} & b_{2} & b_{3}\\ c_{1} & c_{2} & c_{3} \end{array}\right]=\left[\begin{array}{ccc} \cos\varphi & 0 & -\sin\varphi \\ 0 & 1 & 0\\ \sin\varphi & 0 & \cos\varphi \end{array}\right]\left[\begin{array}{ccc} 1 & 0 & 0\\ 0 & \cos\omega & -\sin\omega \\ 0 & \sin\omega & \cos\omega \end{array}\right]\left[\begin{array}{ccc} \cos\kappa & -\sin\kappa & 0\\ \sin\kappa & \cos\kappa & 0\\ 0 & 0 & 1 \end{array}\right],$$

The colinear equations taking the boresight misalignments transformation matrix into account can be expressed as Equation (5):

(5) $$\left[\begin{array}{c} X-X_{S}\\ Y-Y_{S}\\ Z-Z_{S} \end{array}\right]=\lambda R_{b}^{m}\left(\varphi ,\omega ,\kappa \right)R_{c}^{b}\left(\varphi^{\prime },\omega^{\prime },\kappa^{\prime }\right)\left[\begin{array}{c} x-x_{0}\\ y-y_{0}\\ -f \end{array}\right]=\;\lambda \left[\begin{array}{ccc} a_{1} & a_{2} & a_{3}\\ b_{1} & b_{2} & b_{3}\\ c_{1} & c_{2} & c_{3} \end{array}\right]\left[\begin{array}{ccc} a_{1}\prime & a_{2}\prime & a_{3}\prime \\ b_{1}\prime & b_{2}\prime & b_{3}\prime \\ c_{1}\prime & c_{2}\prime & c_{3}\prime \end{array}\right]\left[\begin{array}{c} x-x_{0}\\ y-y_{0}\\ -f \end{array}\right],$$

where

(6) $$\left[\begin{array}{ccc} a_{1} & a_{2} & a_{3}\\ b_{1} & b_{2} & b_{3}\\ c_{1} & c_{2} & c_{3} \end{array}\right]=\left[\begin{array}{ccc} \cos\varphi & 0 & -\sin\varphi \\ 0 & 1 & 0\\ \sin\varphi & 0 & \cos\varphi \end{array}\right]\left[\begin{array}{ccc} 1 & 0 & 0\\ 0 & \cos\omega & -\sin\omega \\ 0 & \sin\omega & \cos\omega \end{array}\right]\left[\begin{array}{ccc} \cos\kappa & -\sin\kappa & 0\\ \sin\kappa & \cos\kappa & 0\\ 0 & 0 & 1 \end{array}\right],$$

and

(7) $$\left[\begin{array}{ccc} a_{1}\prime & a_{2}\prime & a_{3}\prime \\ b_{1}\prime & b_{2}\prime & b_{3}\prime \\ c_{1}\prime & c_{2}\prime & c_{3}\prime \end{array}\right]=\;\left[\begin{array}{ccc} \cos\varphi \prime & 0 & -\sin\varphi \prime \\ 0 & 1 & 0\\ \sin\varphi \prime & 0 & \cos\varphi \prime \end{array}\right]\left[\begin{array}{ccc} 1 & 0 & 0\\ 0 & \cos\omega \prime & -\sin\omega \prime \\ 0 & \sin\omega \prime & \cos\omega \prime \end{array}\right]\left[\begin{array}{ccc} \cos\kappa \prime & -\sin\kappa \prime & 0\\ \sin\kappa \prime & \cos\kappa \prime & 0\\ 0 & 0 & 1 \end{array}\right],$$

*x* and *y* denote the image coordinates of a tie point in the image coordinate system; f is the focal length of the camera; *X*, *Y*, *Z* are the coordinates of the ground point corresponding to the image pixel in the object coordinate system; *X_S_*, *Y_S_*, *Z_S_* are the coordinates of the projection center of the camera; *λ* is a scale factor; and $a_{i},b_{i},c_{i}$ are the elements of the rotation matrix $R_{b}^{m}\left(\varphi ,\omega ,\kappa \right)$. Boresight misalignments are denoted by φ′,ω′,k′, and its rotation matrix is denoted by $R_{c}^{b}\left(\varphi^{\prime },\omega^{\prime },k^{\prime }\right)$.

In general, the values of $\varphi^{\prime },\omega^{\prime },k\prime$ are very small. Thus, the matrix of boresight misalignments can be approximated as follows:

(8) $$R_{c}^{b}\left(\varphi^{\prime },\omega^{\prime },\kappa^{\prime }\right)=\left[\begin{array}{ccc} 1 & 0 & -\varphi^{\prime }\\ 0 & 1 & 0\\ \varphi^{\prime } & 0 & 1 \end{array}\right]\left[\begin{array}{ccc} 1 & 0 & 0\\ 0 & 1 & -\omega^{\prime }\\ 0 & \omega^{\prime } & 1 \end{array}\right]\left[\begin{array}{ccc} 1 & -\kappa^{\prime } & 0\\ \kappa^{\prime } & 1 & 0\\ 0 & 0 & 1 \end{array}\right]=\left[\begin{array}{ccc} 1 & -\kappa^{\prime } & -\varphi^{\prime }\\ \kappa^{\prime } & 1 & -\omega^{\prime }\\ \varphi^{\prime } & \omega^{\prime } & 1 \end{array}\right]$$

Equation (5) can be compactly rewritten as:

(9) $$\left[\begin{array}{c} x-x_{0}\\ y-y_{0}\\ -f \end{array}\right]=\;\frac{1}{\lambda }{\left[\begin{array}{ccc} a_{1}\prime & a_{2}\prime & a_{3}\prime \\ b_{1}\prime & b_{2}\prime & b_{3}\prime \\ c_{1}\prime & c_{2}\prime & c_{3}\prime \end{array}\right]}^{-1}{\left[\begin{array}{ccc} a_{1} & a_{2} & a_{3}\\ b_{1} & b_{2} & b_{3}\\ c_{1} & c_{2} & c_{3} \end{array}\right]}^{-1}\left[\begin{array}{c} X-X_{S}\\ Y-Y_{S}\\ Z-Z_{S} \end{array}\right]=\frac{1}{\lambda }\left[\begin{array}{ccc} m_{11} & m_{12} & m_{13}\\ m_{21} & m_{22} & m_{23}\\ m_{31} & m_{32} & m_{33} \end{array}\right]\left[\begin{array}{c} X-X_{S}\\ Y-Y_{S}\\ Z-Z_{S} \end{array}\right],$$

or

(10) $$\left\{\begin{array}{c} x-x_{o}=-f\frac{m_{11}\left(X-X_{S}\right)+m_{12}\left(Y-Y_{S}\right)+m_{13}\left(Z-Z_{S}\right)}{m_{31}\left(X-X_{S}\right)+m_{32}\left(Y-Y_{S}\right)+m_{33}\left(Z-Z_{S}\right)}\\ y-y_{o}=-f\frac{m_{21}\left(X-X_{S}\right)+m_{22}\left(Y-Y_{S}\right)+m_{23}\left(Z-Z_{S}\right)}{m_{31}\left(X-X_{S}\right)+m_{32}\left(Y-Y_{S}\right)+m_{33}\left(Z-Z_{S}\right)} \end{array}\right.,$$

$\;\;m_{ij}$ are the elements in the matrix generated by multiplying the inverse of $R_{b}^{m}$ and $R_{c}^{b}\;\;$.

Take the x, y coordinates of an image tie point as the observations and boresight misalignments φ′,ω′,k′ as the unknowns. Denote the initial values of *x* and *y* by (*x*) and (*y*), respectively, which are calculated from Equation (5), and then the linearization of Equation (10) can be expressed as:

(11) $$\left\{\begin{array}{c} x=\left(x\right)+\frac{\partial F_{x}}{\partial \varphi \prime }\Delta \varphi \prime +\frac{\partial F_{x}}{\partial w\prime }\Delta w\prime +\frac{\partial F_{x}}{\partial \kappa \prime }\Delta \kappa \prime \\ y=\left(y\right)+\frac{\partial F_{y}}{\partial \varphi \prime }\Delta \varphi \prime +\frac{\partial F_{y}}{\partial w\prime }\Delta w\prime +\frac{\partial F_{y}}{\partial \kappa \prime }\Delta \kappa \prime \end{array}\right.$$

Take the V as the correction value, then the error equation can be established as:

(12) V=AB−L,

where

$$\begin{array}{cc} \begin{array}{c} V={\left[v_{x},v_{y}\right]}^{T},\\ B={\left[∆\varphi \prime ,∆\omega \prime ,∆\kappa \prime \right]}^{T},\\ L=[l_{x},l_{y}{]}^{T}=[x-\left(x\right),y-\left(y\right){]}^{T}, \end{array} & A=\left[\begin{array}{ccc} \frac{\partial F_{x}}{\partial \varphi \prime } & \frac{\partial F_{x}}{\partial w\prime } & \frac{\partial F_{x}}{\partial \kappa \prime }\\ \frac{\partial F_{y}}{\partial \varphi \prime } & \frac{\partial F_{y}}{\partial w\prime } & \frac{\partial F_{y}}{\partial \kappa \prime } \end{array}\right] \end{array}$$

A normal equation can be obtained by the indirect adjustment principle:

(13) $$A^{T}WAB=A^{T}WL,$$

where *W* is the weight matrix of the observations, which can be assigned a unit matrix, because all observations are assumed to have the same accuracy. Therefore, the corrections of the boresight misalignments can be calculated as follows:

(14) $$B={\left(A^{T}A\right)}^{-1}A^{T}L$$

It is worth pointing out that, while the three rotation angles φ,ω,k can be derived from data of the POS system, the angular elements it provides are heading and pitch, which do not comply with rotation angles used in photogrammetry. Several methods were developed to transform these two sets of angles. We adopted the method proposed by Zhao et al. [59], in which the compensation matrix is not required.

The next step is to calculate the image coordinates $\left(x^{\prime },y^{\prime }\right)$ corresponding to the virtual control points using Equation (5) with the corrected boresight misalignments and other exterior orientation elements of that image.

Assume there are n tie points, for a given point $\left(x_{i},y_{i}\right)$, and calculate the distance between it and its corresponding $\left(x_{i}^{\prime },y_{i}^{\prime }\right)$. Then, average error $\left(Ex,Ey\right)$ and RMSE (Root Mean Square Error) $\left(Rx,Ry\right)$ can be calculated by the following formula:

(15) $$\left\{\begin{array}{c} \begin{array}{c} dx_{i}=x_{i}-x_{i}^{\prime }\\ dy_{i}=y_{i}-y_{i}^{\prime }\\ Ex=\frac{1}{n}\overset{n}{\underset{i=1}{\sum }}\left|dx_{i}\right| \end{array}\\ Ey=\frac{1}{n}\overset{n}{\underset{i=1}{\sum }}\left|dy_{i}\right|\\ Rx=\sqrt{\frac{1}{n}\overset{n}{\underset{i=1}{\sum }}{\left(dx_{i}\right)}^{2}}\\ Ry=\sqrt{\frac{1}{n}\overset{n}{\underset{i=1}{\sum }}{\left(dy_{i}\right)}^{2}} \end{array}\right.$$

These errors plus the maximum iterations determine when the iteration will be stopped: if all four errors remain stable in the iteration, or maximum iterations have been reached, then the iteration stops and the boresight misalignments are obtained.

## 3. Results

### 3.1. Results of Tie Points Detection and Matching

**Figure 8:**
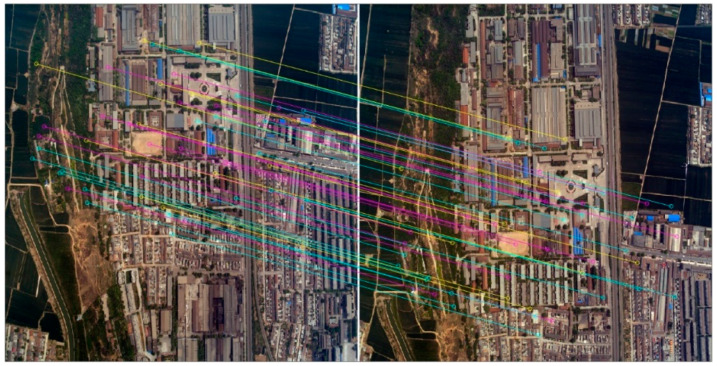
Partial view of the tie points obtained by SIFT (showing only 50 tie points).

**Figure 9:**
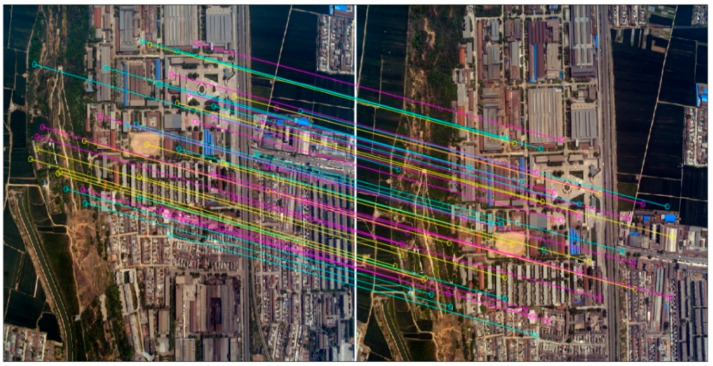
Partial view of the tie points obtained by SURF (showing only 50 tie points).

Tie points detection and matching by SURF is described in detail in Section 2.2. SIFT (Scale Invariant Feature Transform) algorithm was adopted for the sake of comparison. Figure 8 and Figure 9 show the results by SIFT and SURF detection and matching, respectively. Table 3 lists the performance of the two algorithms, including the number of tie points detected and matched, the time cost, and the accuracy.

As shown in Table 3, SURF and SIFT have roughly the same tie points offset and matching accuracy. Both can detect and match enough tie points for direct georeferencing purposes. In the experiment, the SURF algorithm detected and matched 1036 tie points, among which 832 are correct, while the SIFT algorithm extracted 2259 tie points in total, and 1830 are correct. However, the time cost of the SIFT algorithm is significantly higher than the SURF algorithm, which coincides with previous comparison studies such as those carried out by Mistry and Banerjee [60]. Thus, we use the SURF algorithm for tie points detection and matching.

**Figure 10:**
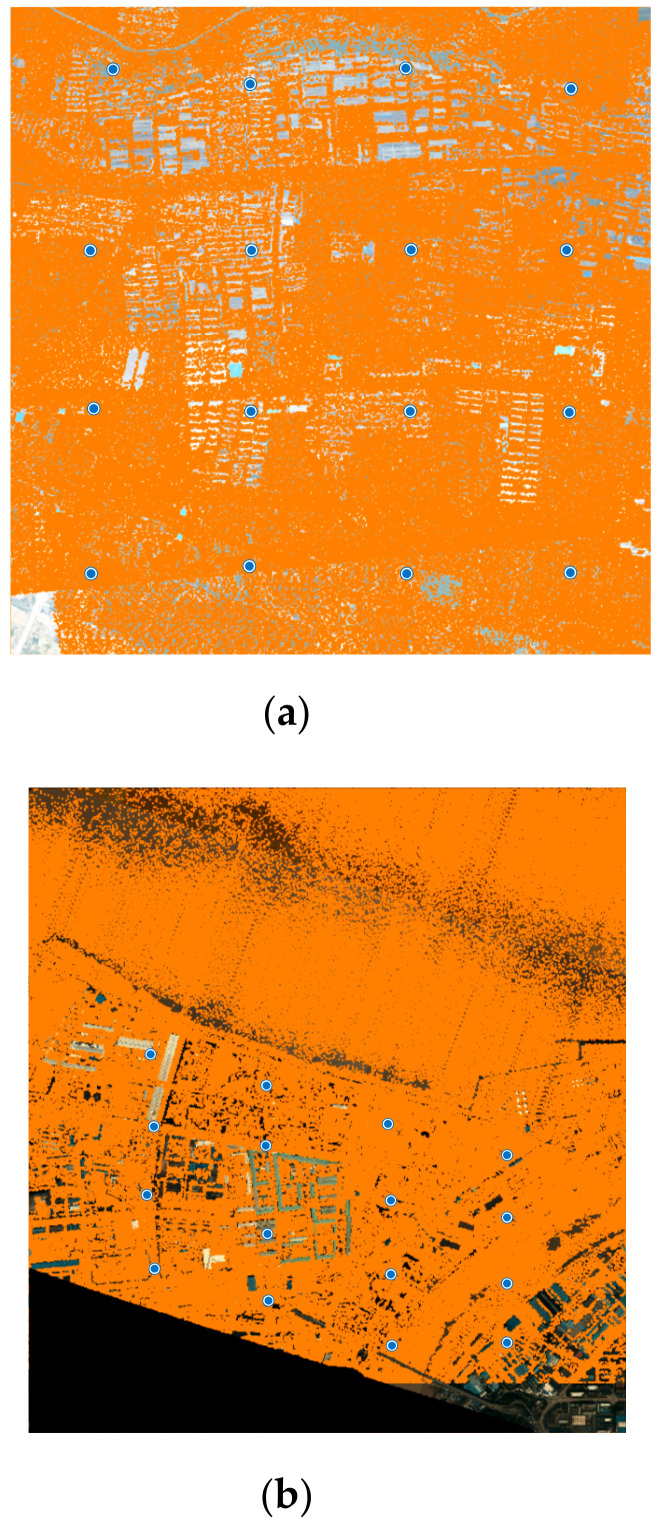
Virtual control points (16 selected VCPs): (**a**) Dataset 1; and (**b**) Dataset 2.

Space intersection was performed to the tie point dataset, from which object points corresponding to tie points were obtained, which in turn formed the candidates of virtual control points. Because redundant tie points were detected and matched thanks to the SURF algorithm, so does the object points. A VCP selection procedure described in Section 2.2.3. was applied to select virtual control points. Figure 10 shows the locations of VCPs of the two datasets. Since the upper half area of Dataset 2 covers a beach area, tie points extracted were error prone, and preference was given to the tie points distributed in the lower part of the dataset when space intersection was performed.

### 3.2. Results of Direct Georeferencing by Boresight Misalignments Calibration

As described in Section 2.2.4, boresight misalignments were calibrated with VCPs in an iterative manner, followed by direct georeferencing for the two image datasets. The effectiveness of boresight misalignments calibration can be evaluated by assessing the accuracy of the georeferencing images before and after the misalignments were corrected. Other visual inspections of the effectiveness include the check of the mosaiced two adjacent images to see if continuous linear features such as roads are seamlessly mosaiced, and the registration of the point cloud and the georeferenced images. Since accuracies were achieved for the two datasets, quantitative evaluations were given to the first dataset only to avoid a lengthy repetitive analysis. Figures for visual inspection are provided for the second dataset.

The misalignments of the first datasets and their relationship with the number of images used for calibration are tabulated in Table 4. As shown in the table, when the number of images is small and the images are distributed in less strips, the calibration results are unstable; when four strips are used, and the number of images greater than 16, calibration results tend to be stable.

**Figure 11:**
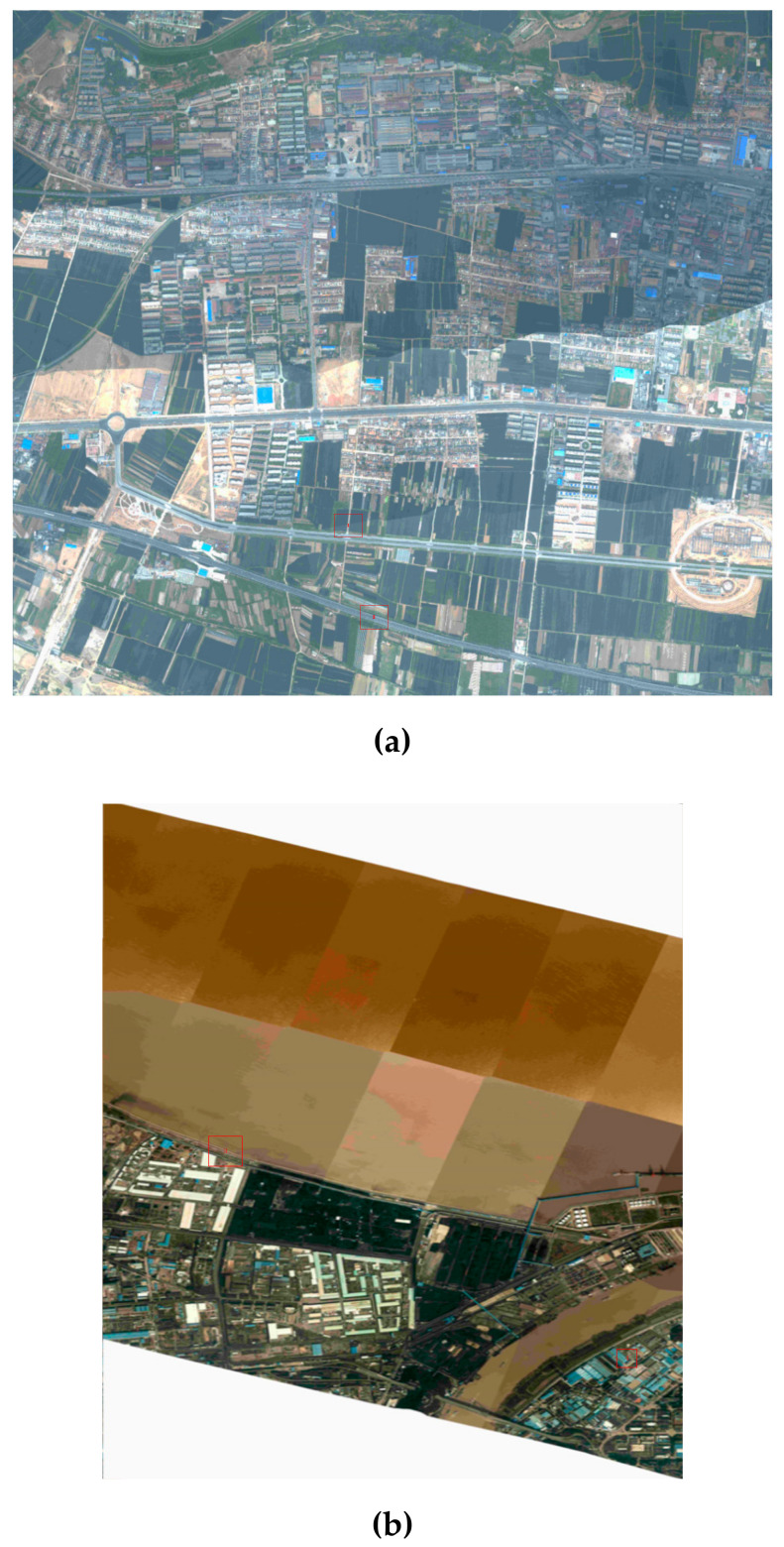
A panoramic view of the DOM generated with boresight misalignments calibration: (**a**) Dataset 1; and (**b**) Dataset 2.

The mosaic images of the two datasets after boresight misalignments calibration are shown in Figure 11.

Eighteen check points (their distribution is shown in Figure 7) collected by RTK (Real-Time Kinematic) technique were used to evaluate the planar accuracy of the georeferenced images of the first image dataset. The results are tabulated in Table 5. Six methods published in literature were selected to compare the performance with ours, which was measured by Root Mean Squared Error (RMSE) of the georeferenced images (see Table 6)

As shown in Table 5, the RMSE of the first dataset is approximately 0.39, 0.51, and 0.64 m in X offset, Y offset, and XY offset, respectively, decreasing 2–3 times compared with those before boresight misalignments calibration.

Table 5 and Table 6 show that some methods achieve higher accuracy than ours. This is mainly due to the fact that tie points or LiDAR control points are extracted manually by the methods in [35,39,40,45]. While the method in [16] perform two steps before extracting the LiDAR control points: the determination of the initial coordinates of the photogrammetric point cloud and the registration of the photogrammetric point cloud to the reference LiDAR surface using least squares surface matching method.

**Figure 12:**
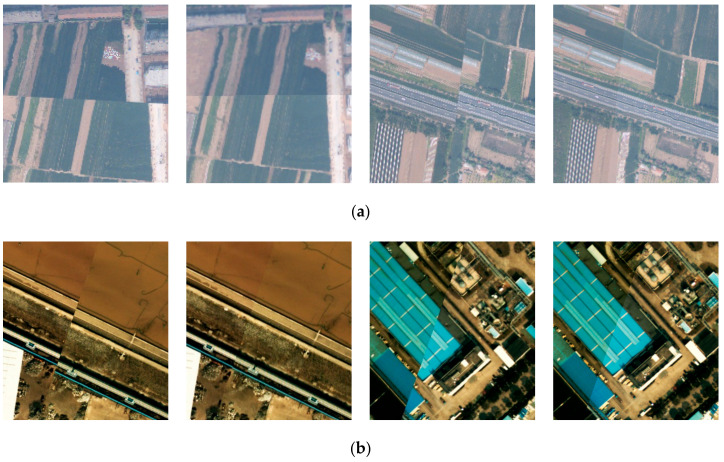
Local area of georeferenced images before and after boresight misalignments calibration: (**a**) Dataset 1; and (**b**) Dataset 2.

**Figure 13:**
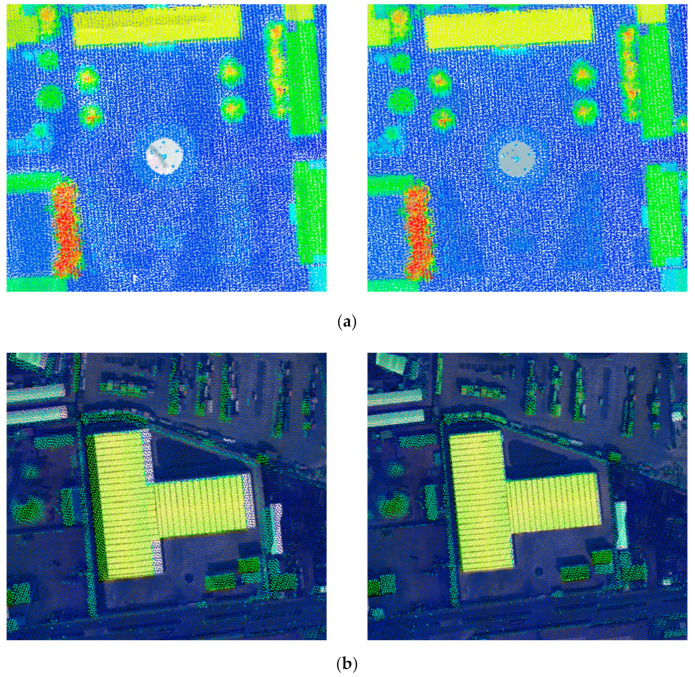
Overlapping LiDAR points with georeferenced images: (**a**) Dataset 1; and (**b**) Dataset 2.

Figure 12 provides the visual check for the plane accuracy of the geo-referenced image before and after boresight misalignments calibration (red rectangle in Figure 11), while Figure 13 shows the result of LiDAR point cloud overlapped with georeferenced images. Together with the quantitative accuracy evaluation, it is obvious that the proposed method is effective, and has a great value in case of specific applications such as rapid response where no control points could be collected and where relatively lower accuracy is required.

## 4. Discussions

The most important step in boresight misalignments calibration is the establishment of correspondence between tie points and control information. Tie points detection and VCPs selection are two core stages in this study. In the former stage, tie points, which are the basic input data to perform the method, were detected and matched by SURF algorithm, which is a mature feature detection and matching algorithm. Since the image has been positioned using the initial EOEs and IOEs provided by the POS system, only the feature points in the center *S*-scale window where the feature points are located are paired. This step can significantly improve the extraction efficiency of tie points. In the later stage, we design an automated tie points selection procedure to perform various assessments to ensure a high quality of point selection from the large numbers of points. These assessments involve planarity tests to select points located over planar surfaces, slope tolerances calculation to avoid the selected points are located in areas with large vertical differences, and reliability checking to ensure that the selected points have enough redundancy for blunder detection.

**Figure 14:**
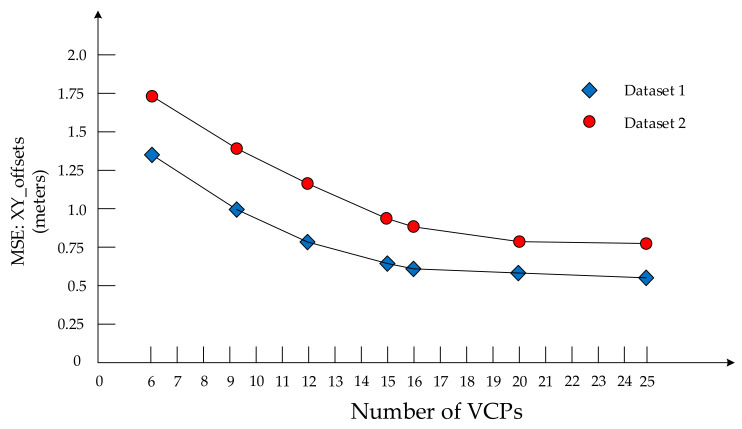
The influence of the number of VCPs.

The number of VCPs used and the impact on the calibration results were studied as well. Figure 14 shows the accuracy curve of georeferenced images versus the number of VCPs. The curve tends to be horizontal when the number of VCPs is larger than 16. In addition, the distribution of tie points is an equally important factor. An ideal distribution pattern requires that all the tie points be distributed evenly in the whole surveying area.

## 5. Conclusions

In this paper, a new direct georeferencing method based on boresight misalignments calibration is presented. We validated this method with two different camera systems. Experimental results show that the planar accuracy of the resultant georeferenced images after boresight misalignments calibration is increased significantly compared to those produced directly by using the initial orientation elements from POS data. Moreover, the georeferenced images produced by the proposed method registered the LiDAR points much better. The proposed method allows for boresight misalignments calibration in areas where new calibration fields cannot be established or where rapid response is required in situations such as earthquake relief.

Theoretically, the method is also applicable when the point cloud is not collected simultaneously with the images. Such point cloud can be collected previously by a LiDAR system or generated from other means such as stereo-image matching, only if the images are collected with auxiliary of a POS system. However, such experiments were not conducted in the study and were left for further study. Meanwhile, because the boresight misalignments were contained in the collinear equations as an extra rotation in addition to the rotation caused by the three angular elements of the exterior orientation elements when a virtual control point was projected onto the image space, it is unlikely that other sources of errors can be included as well. Other adjustment models may overcome this shortcoming, but it remains for further studies.

## Figures and Tables

**Table 1:** Technical parameters of Dataset 1.

**Flight Information**	**Target Area**	**Max Flight Height**	**Min Flight Height**	**Number of Flights**	**Number of Images**
	Xi’an, China	1450 m	1387 m	4	49
**LiDAR Points**	**Sensor**	**Point Cloud Density**	**FOV**	**Size of Area**	**Average Overlap**
	Leica ALS60	1.9/m^2^	45°	11 km^2^	48%
**Aerial Images**	**Type of Camera**	**Pixel Size**	**Focal Length**	**Forward Overlap**	**Side Overlap**
	RCD105	0.0068 mm	60 mm	70%	45%

**Table 2:** Technical parameters of Dataset 2.

**Flight Information**	**Target Area**	**Max Flight Height**	**Min Flight Height**	**Number of Flights**	**Number of Images**
	Ningbo, China	2397 m	2304 m	3	27
**LiDAR Points**	**Sensor**	**Point Cloud Density**	**FOV**	**Size of Area**	**Average Overlap**
	Leica ALS70	0.60/m^2^	45°	15 km^2^	20%
**Aerial Images**	**Type of Camera**	**Pixel Size**	**Focal Length**	**Forward Overlap**	**Side Overlap**
	RCD30	0.006 mm	53 mm	60%	30%

**Table 3:** Precision parameter of tie points extraction experiment.

Algorithm	Tie Points Offset	Time Cost	Number of Tie Points	Correct Tie Points	Accuracy
SURF	1–3 pixels	17 s	1036	832	80.3%
SIFT	1–3 pixels	329 s	2259	1830	81.0%

**Table 4:** Misalignments (in degrees) calibrated by different numbers of images.

Number of Strips	Number of Images	φ′	ω′	κ′
2	2	0.3973	0.6913	0.3122
	4	−0.3022	0.5170	0.1613
	6	−0.3014	0.5640	0.3399
	8	−0.3719	0.5918	0.2766
	10	−0.3519	0.6018	0.2966
3	3	−0.3767	0.6529	0.3835
	6	−0.3255	0.5621	0.3029
	9	−0.3240	0.5587	0.2987
	12	−0.3320	0.5301	0.2758
	15	−0.3120	0.5605	0.2951
4	4	−0.3552	0.4682	0.3967
	8	−0.3417	0.5592	0.2964
	16	−0.3223	0.5619	0.2957
	20	−0.3221	0.5615	0.2962
	24	−0.3222	0.5616	0.2958

**Table 5:** Planar accuracy of the georeferenced images.

Residual Errors (m)	Georeferenced Images Before Boresight Misalignments Calibration			Georeferenced Images After Boresight Misalignment Calibration		
	dX	dY	dXY	dX	dY	dXY
Average value	1.0241	2.9382	3.1115	0.2398	−0.3511	0.5065
Max value	1.7276	5.4239	5.6924	0.8113	1.1249	1.3869
RMSE	0.8943	1.6423	1.8700	0.3915	0.5135	0.6459

**Table 6:** Comparison of the performance of our method and six others by RMSE along x and y axes, which are denoted RMSEx and RMSEy, respectively. RMSExy = $\sqrt{{\mathrm{RMSEx}\;}^{2}+{\mathrm{RMSEy}}^{2}}\;$.

Method from Literature Numbered	RMSE_x_ (m)	RMSE_y_ (m)	RMSE_xy_ (m)
[16]	0.11	0.05	0.12
[35]	0.35	0.45	0.57
[36]	0.25	0.16	0.30
[39]	0.45	1.18	1.26
[40]	0.33	0.33	0.47
[45]	0.35	0.77	0.85
Ours	0.39	0.51	0.64
